# Multidimensional individualised Physical ACTivity (Mi-PACT) – a technology-enabled intervention to promote physical activity in primary care: study protocol for a randomised controlled trial

**DOI:** 10.1186/s13063-015-0892-x

**Published:** 2015-08-28

**Authors:** Oliver J. Peacock, Max J. Western, Alan M. Batterham, Afroditi Stathi, Martyn Standage, Alan Tapp, Paul Bennett, Dylan Thompson

**Affiliations:** Department for Health, University of Bath, Bath, BA2 7AY UK; Health and Social Care Institute, Teesside University, Middlesbrough, UK; Bristol Business School, University of the West of England, Bristol, UK

**Keywords:** Physical activity, Sedentary behaviour, Technology, Feedback, Self-monitoring, Behaviour change, Primary care, Health promotion

## Abstract

**Background:**

Low physical activity is a major public health problem. New cost-effective approaches that stimulate meaningful long-term changes in physical activity are required, especially within primary care settings. It is becoming clear that there are various dimensions to physical activity with independent health benefits. Advances in technology mean that it is now possible to generate multidimensional physical activity ‘profiles’ that provide a more complete representation of physical activity and offer a variety of options that can be tailored to the individual. Mi-PACT is a randomised controlled trial designed to examine whether personalised multidimensional physical activity feedback and self-monitoring alongside trainer-supportive sessions increases physical activity and improves health outcomes in at-risk men and women.

**Methods/Design:**

We aim to recruit 216 patients from within primary care aged 40 to 70 years and at medium or high risk of cardiovascular disease and/or type II diabetes mellitus. Adopting an unequal allocation ratio (intervention: control) of 2:1, participants will be randomised to one of two groups, usual care or the intervention. The control group will receive usual care from their general practitioner (GP) and standardised messages about physical activity for health. The intervention group will receive physical activity monitors and access to a web-based platform for a 3-month period to enable self-monitoring and the provision of personalised feedback regarding the multidimensional nature of physical activity. In addition, this technology-enabled feedback will be discussed with participants on 5 occasions during supportive one-to-one coaching sessions across the 3-month intervention. The primary outcome measure is physical activity, which will be directly assessed using activity monitors for a 7-day period at baseline, post intervention and at 12 months. Secondary measures (at these time-points) include weight loss, fat mass, and markers of metabolic control, motivation and well-being.

**Discussion:**

Results from this study will provide insight into the effects of integrated physical activity profiling and self-monitoring combined with in-person support on physical activity and health outcomes in patients at risk of future chronic disease.

**Trial registration:**

ISRCTN18008011 Trial registration date: 31 July 2013

## Background

Physical inactivity has substantial effects on global health and an increase in activity at the population level would have considerable impact on the future burden of chronic disease [1]. In the United Kingdom, the Department of Health recently began a national programme (NHS Health Check) that aims to reduce chronic disease by identifying adults who are at increased risk and offering them personalised advice and support to lower their risk [2]. Physical activity has the potential to increase the success of such initiatives, but existing interventions in primary care have met with limited success and typically small improvements are not maintained [3]. New cost-effective approaches that stimulate meaningful long-term changes in physical activity are required and this is especially important in those identified as at risk of cardiovascular disease and type II diabetes mellitus.

To date, physical activity has typically been captured and recommended in unidimensional terms (e.g. 150 minutes of moderate intensity physical activity per week) [4]. Physical activity is a much more heterogeneous behaviour than this approach implies, with various dimensions known to have clear biological and health benefits [5]. Indeed, past work shows that it is quite possible for an individual to score highly in one dimension of physical activity but low in another, while only very few people score consistently across all physical activity metrics [6]. This observation is a problem because people who focus on a single physical activity descriptor may form incomplete or inaccurate conclusions about the appropriateness of their behaviour. For example, many forms of structured physical activity have only a modest impact on overall energy expenditure [7]. Weight loss is critical to some health outcomes, or an outcome in itself, and it will be important for individuals aiming to lose weight (or prevent weight regain after substantial loss) to understand which aspects of physical activity have the largest thermogenic effect. In this specific scenario, a multidimensional approach will help people incorporate novel activity within the context of their existing behaviour such that the net effect on total energy expenditure is maximised [7, 8]. Clearly, a multidimensional profile will provide greater insight, awareness, and deeper understanding than the reliance on more unidimensional feedback; enabling people to take greater responsibility for managing their physical activity.

In addition to providing a more accurate reflection of physical activity, the diverse options associated with a multidimensional physical activity profile create an exploitable social marketing opportunity. The marketing of structured and informational feedback and the provision of personally relevant and attainable physical activity options is potentially a key step in supporting an individual’s satisfaction of autonomy and competence [9, 10]. When people experience a sense of self-endorsed and choicefully enacted behaviour this tends to improve the quality of their motivation and sustain engagement in physical activity [9, 10]. Indeed, the provision of more comprehensive and revealing feedback on behaviour should also raise awareness and support the formation of implementation intentions (i.e. where, when and how to act); increasing goal attainment and habit formation [11].

New technologies, which include wearable devices and web-based applications, enable self-monitoring of physical activity and create opportunities for the provision of personalised feedback regarding the multidimensional nature of physical activity [5]. This type of individually tailored feedback tends to be more effective than generic messages about physical activity [12, 13]. Moreover, feedback and self-monitoring in combination with specific goal setting are acknowledged as key constituents of successful behavioural interventions [14–16]. In order to exploit the opportunities for physical activity monitoring and multidimensional physical activity profiling, we developed a website-based application for linking physical activity data with informational feedback; creating an interface for self-monitoring, specific planning and trainer interaction, that collectively forms the basis for the present trial. This was informed by prior work, in which we generated visualisations for the presentation of integrated physical activity profiles and demonstrated that patients at medium or high risk of chronic disease found this feedback to be informative, understandable and motivating [17]. In addition, while patients reported feeling confident in using technology and feedback for self-monitoring physical activity, it was identified that supplementary in-person guidance may further support behaviour change.

The Multidimensional individualised Physical ACTivity (Mi-PACT) study is a randomised controlled trial with the following objectives: (1) to examine whether personalised multidimensional physical activity feedback and self-monitoring using a web-based platform alongside in-person advice supports an increase in physical activity in men and women at risk of future chronic disease; and (2) to examine whether this change is sufficient to generate meaningful weight loss and/or improved metabolic control and reduced risk.

## Methods

### Trial design

**Fig. 1:**
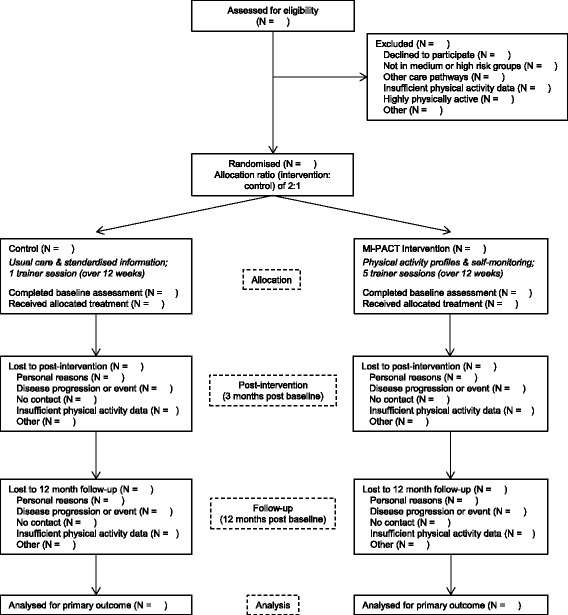
Flow of participants through the study. Patients identified as ‘at risk’ and who volunteer will be assessed for eligibility via telephone screen and attendance at a baseline assessment clinic. Eligible people will be randomised to either the control (usual care) or intervention (Mi-PACT) group by concealed minimisation. Follow-up assessments will take place post intervention at 3 and 12 months

In Mi-PACT, patients at risk of cardiovascular disease or type II diabetes mellitus are randomly assigned to receive usual care (control group) or technology-enabled multidimensional physical activity feedback (intervention group). Figure 1 illustrates the course of progress through the study consistent with current Consolidated Standards of Reporting Trials (CONSORT) guidelines for reporting randomised trials [18]. The trial described herein was approved by the National Health Service (NHS) South West 3 Research Ethics Committee with an allocated reference number: 13/SW/0179. The project was subsequently registered as a current controlled trial (ISRCTN18008011).

### Participants/eligibility

We will recruit men and women treated in primary care aged 40–70 years at medium (≥10 and < 20 %) or high (≥20 %) risk of cardiovascular disease and/or type II diabetes mellitus. Risk will be calculated from clinical data using well-established prediction algorithms for estimating a person’s 10-year risk of developing cardiovascular disease (QRISK®2) [19] and diabetes mellitus (QDiabetes®) [20] available at QResearch® [21]. As our focus is on prevention, people with existing coronary heart disease, chronic kidney disease (stages 3–5), diabetes mellitus, stroke, heart failure and peripheral arterial disease (as they will be managed via existing care pathways) will be excluded. The Physical Activity Level (PAL) is a standard objective method of expressing total daily energy expenditure in multiples of resting metabolic rate. Individuals will be excluded with an average daily PAL > 2.0, which has been categorised in a report of a joint FAO/WHO/UNU expert consultation as representing a highly active lifestyle [22]. In addition, individuals will be excluded for whom sufficient baseline physical activity data is not available. To be eligible, individuals will need at least 6 valid monitoring days (including both a Saturday and Sunday) [23]. A valid day will require at least 80 % of data for a given 24-hour period. See Table 1 for a complete list of eligibility criteria.

**Table 1:** Inclusion and exclusion criteria for Mi-PACT

Inclusion criteria
• Able to give informed consent to participate in the study
• Aged between 40–70 years
• Recruited from within primary care
• At medium (≥10 and < 20 %) or high (≥20 %) risk of cardiovascular disease and/or type II diabetes mellitus
Exclusion criteria
• People with existing coronary heart disease, chronic kidney disease, (stages 3–5), diabetes mellitus, stroke, heart failure and peripheral arterial disease
• Resting blood pressure greater than 180/100 mmHg
• Body mass index greater than 40 kg/m^2^
• Currently pregnant
• Currently taking any medications that may affect weight loss
• Not fluent in English
• Currently participating in another research trial or lifestyle supportive intervention
• People reporting a recent (in the last 6 months) shift (>5 %) in body mass or large change in habitual lifestyle
• Individuals unable to change their physical activity (e.g. through disability) or individuals who already lead highly physically active lifestyles (PAL > 2.0)
• Insufficient baseline physical activity data (<6 valid monitoring days including a Saturday and Sunday)
• People with terminal illness and anyone who, in their general practitioner’s opinion, has other co-morbidities which would prevent engagement with the intervention

*PAL* Physical Activity Level

### Study procedures

#### Recruitment

Patients will be recruited in Bath and North East Somerset and Wiltshire. We aim to recruit at least five general practices with variety in terms of socioeconomic status according to National General Practice Profiles and the English Indices of Deprivation [24, 25]. We will recruit potentially eligible participants via two routes. Firstly, individuals in medium or high-risk groups (based on existing risk-score information in patient notes) will be identified by searching practice databases. Secondly, because not all people will have risk information in their records, individuals at potentially increased risk will be identified (people with a body mass index (BMI) > 30 kg/m^2^ in combination with a total cholesterol level between 5.5 and 7.5 mmol/L and/or blood pressure > 140/90 mmHg). This is consistent with Health Check criteria, and clinical data from baseline assessments will be used to verify that individuals are in medium or high-risk groups, using QRISK®2 and QDiabetes® algorithms [19, 20]. Potentially eligible participants will then be approached by a letter from their general practitioner (GP). The recruitment letter will emphasise that participation is entirely voluntary and that individuals will be free to withdraw at any time without any impact on their health care provision. A study information sheet and sample consent form will be included with the letter so that patients have time to consider their participation. The invitation letter will include a free post reply slip for the patient to return to the research team indicating whether or not they wish to learn more about the study. Individuals who return positive replies will then be contacted by the research team. Those not wishing to participate will not be contacted again.

#### Participant screening and baseline assessment

A research nurse will first conduct a telephone screen to re-confirm eligibility. In addition, information regarding marital status, ethnicity, smoking status, profession and education will be recorded. All potentially eligible participants interested in taking part will be invited to attend a 60-minute baseline assessment clinic following an overnight fast. At baseline clinics, the research nurse will further explain the nature of the study and answer any questions. Individuals who agree to participate following the briefing will be provided with an informed consent form, indicating their full understanding of the study and their protected rights for confidentiality and withdrawal from the study without giving a reason. For those providing written informed consent, concomitant medications and relevant clinical history will be recorded and a questionnaire pack will be issued for completion within clinic. Thereafter, the nurse or researcher will measure each individual’s blood pressure, weight, height, waist circumference and take a blood sample. During the clinic, individuals will receive an activity monitor with oral and written instruction for use and will be provided with a freepost envelope for returning monitors to the research team. For those participants who choose to opt-in, a visit to the University of Bath will be arranged for the assessment of body composition via dual energy X-ray absorptiometry. Blood results will be shared with each patient’s GP. Patients with a fasting blood glucose of > 7.0 mmol/l at this stage will be excluded from further participation as this will likely initiate further testing by the GP and is indicative of probable diabetes mellitus. People successfully completing activity monitoring who are not highly physically active and are in medium-risk or high-risk groups (confirmed via recalculated risk scores using baseline measurements and the results of blood tests) will be eligible for randomisation.

#### Randomisation/allocation

Eligible people will be randomised to one of two groups. We opt for an unequal allocation ratio (intervention: control) of 2:1, primarily to increase our experience with and amount of information on the new intervention [26] (the small loss of precision with unequal allocation only increases the total *N* required by 20). Participants will be allocated remotely by the trial statistician by concealed minimisation [27], providing balance across the trial arms for sex (male/female), age group (40–59 and 60–70 years), general practice, risk (medium/high) and PAL < 1.75 or ≥ 1.75. Although individual patients are the unit of randomisation, we believe that the threat of contamination within a practice is largely theoretical, especially given that the intervention is personalised.

#### Follow-up assessment

Follow-up data collection will take place post-intervention at 3 and 12 months and will include the same measurements completed during baseline assessment. Participants who opt-in for the assessment of their body composition at baseline will be offered repeat dual-energy X-ray absorptiometry scans at follow-up clinics. Participants will receive a £50 voucher for completing all assessments.

#### Usual care (control)

Participants allocated to the control group will receive usual care by their GP. Any care that they receive in relation to supporting changes in weight or physical activity will be documented through self-report and examination of practice records at assessment clinics. Hence, the trial will assess effectiveness over-and-above existing ‘usual care’ alternatives. In order to standardise exposure to healthcare professionals and content, this group will attend a 20-minute meeting with a lifestyle coach at their GP practice following their baseline assessment (i.e. week 0). At this session, participants will receive standardised information (including printed materials and links to Internet-based resources) regarding cardiovascular disease and type II diabetes mellitus, the potential benefits of physical activity on reducing ‘risk’, current physical activity guidelines and ideas about getting more physically active. Standardised information and messages will be consistent with other print and Internet-based resources available in 2014 (for example, Department of Health: Change4Life [28], NHS Choices: Live Well [29]) and include reference to local opportunities where applicable.

#### Intervention: overview

Mi-PACT is a complex intervention or ‘treatment package’ involving multiple components [30]. The intervention content and iterative web-based platform was developed by the project team and drew heavily on our formative research involving the generation of novel integrated physical activity profiles [4, 6]. The content was further informed by our prior qualitative research with healthcare professionals and patients at risk of future chronic disease (reflecting the intended user group) in the development and evaluation of innovative ways of presenting personalised multidimensional physical activity feedback that is informative, understandable and motivating [17]. The intervention is described in accordance with TIDieR (Template for Intervention Description and Replication) guidance relevant to protocols of trials [31].

#### Intervention: overarching theoretical framework

Social marketing represents an attractively designed ‘customer proposition’ that has been developed and communicated using marketing principles to align with the self-interests of participants and in exchange for behaviour which benefits them and society as a whole [32]. This is where a multidimensional physical activity profile creates a clear social marketing opportunity. Indeed, the marketing of structured and informational feedback coupled with a menu of achievable physical activity options may facilitate greater empowerment (or autonomous engagement) via support of an individual’s satisfaction of autonomy and competence [9]. When people experience autonomy and competence in their treatment, they experience greater volitional engagement and are more likely to persist in desirable health behaviours [9, 10]. In promoting the formation of new habits, implementation intentions are specific plans regarding where, when, and how to act, and have been found to increase goal attainment [11] and predict participation in physical activity [33]. A multidimensional profile that supports the formation of implementation intentions as feedback is more revealing and plans may be considered within the context of an individual’s existing behaviour. Indeed, the combination of implementation intentions and autonomous behaviour has been shown to result in greater goal achievement [34] and represent theoretical constructs that are inherent within the marketing of integrated physical activity profiles.

#### Intervention: physical activity profiling and the Mi-PACT platform

The Mi-PACT technology consists of a Bodymedia Core physical activity monitor (BodyMedia, Inc., Pittsburgh, PA, USA) and a web-based application or ‘platform’ developed in collaboration with Ki Health Innovation Ltd. (Surrey, UK) Information graphics used for the presentation of data within the web-based platform were initially developed alongside graphic designers (Information is Beautiful, IIB Studio, London, UK). Draft designs were subsequently refined by the research team based upon the findings of in-depth qualitative interviews in patient groups and healthcare professionals [17]. Overall, patients preferred simple messages rather than more complex or abstract visualisations [17]. However, as there is unlikely to be a definitive design solution to meet the needs of everyone, and to provide some choice, the Mi-PACT platform includes alternative graphical formats for displaying the same data.

**Fig. 2:**
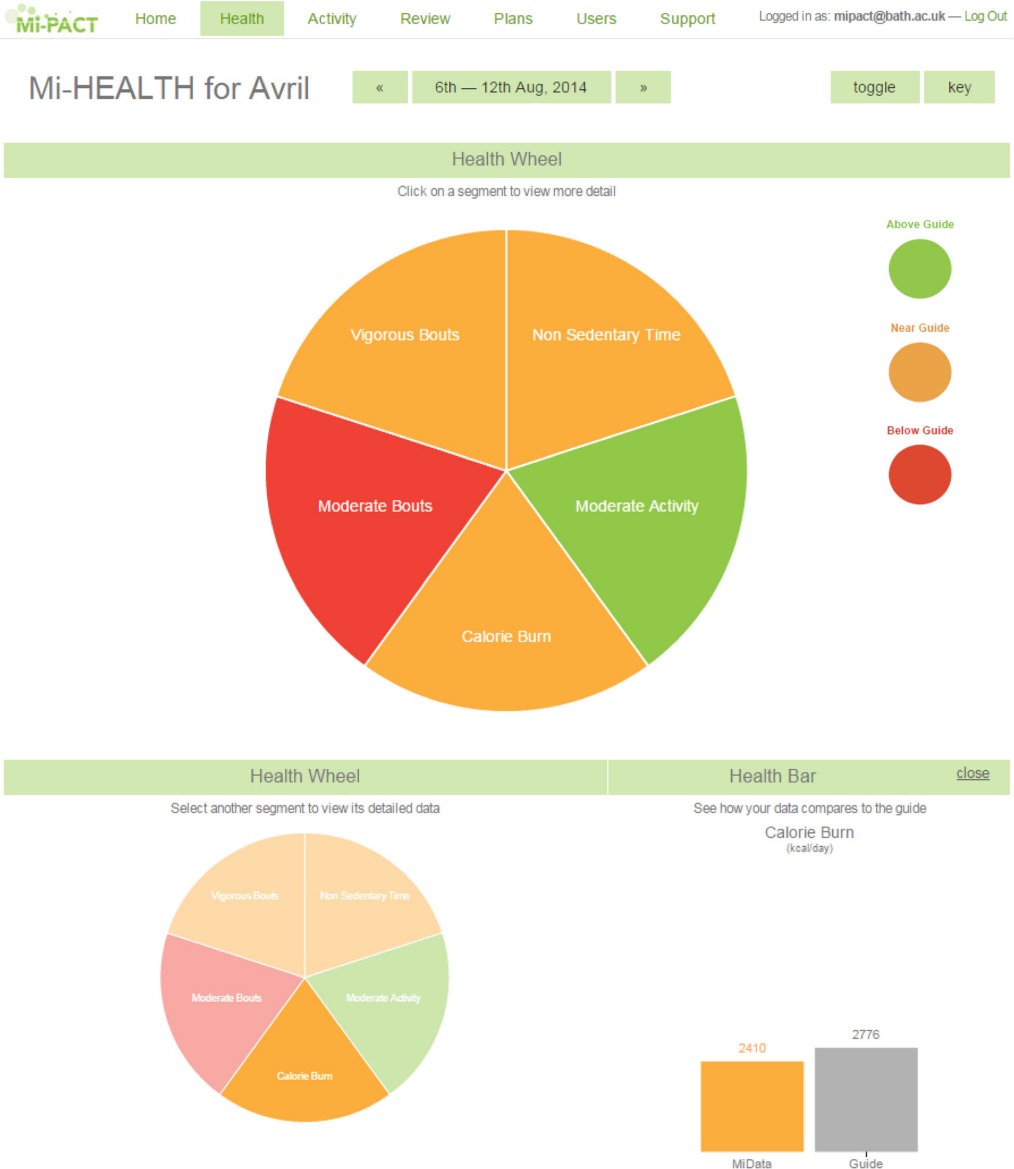
Platform screenshot displaying the integrated physical activity profile across the different physiologically-important dimensions (expressed relative to guidelines)

The integrated physical activity profile captures physical activity across different physiologically important and mutually independent dimensions [6]. Data are depicted in a simple wheel format using a traffic light colour-coding system as an index of attainment (Fig. 2). These data are also presented as colour-coded bars relative to guidelines; allowing an expression of magnitude for each dimension. Each participant’s profile captures 5 different dimensions of their behaviour: (1) overall energy expenditure, (2) time engaged in moderate-to-vigorous physical activity accumulated on a minute-by-minute basis, (3) time engaged in moderate-to-vigorous physical activity accumulated in bouts of at least 10 minutes, (4) time engaged in vigorous intensity activity accumulated in bouts of at least 10 minutes and (5) participation in non-sedentary activity as a proportion of the waking day.

**Fig. 3:**
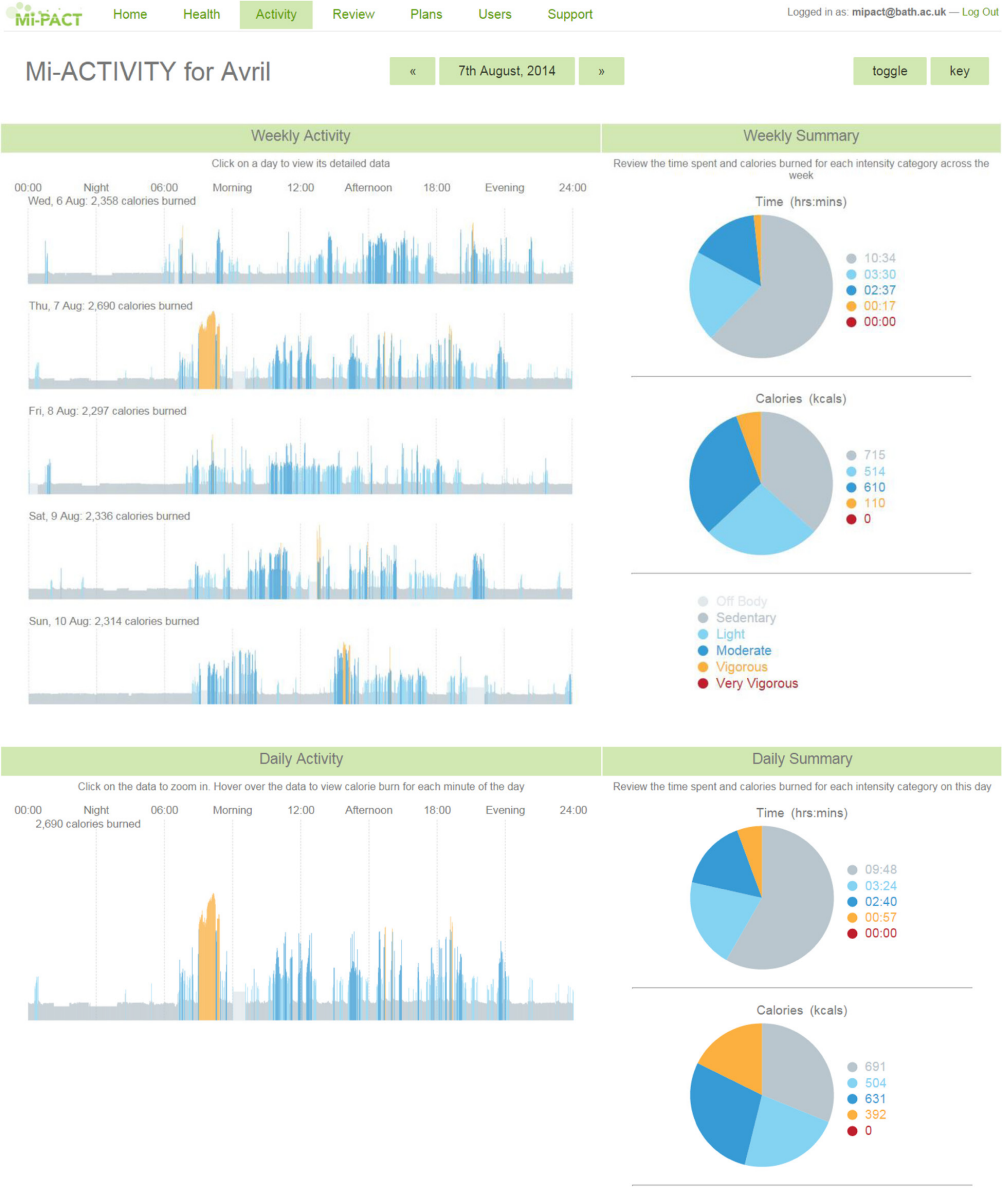
Platform screenshot depicting minute-by-minute energy expenditure and both time spent and energy expended in different intensity thresholds (as daily or weekly summaries)

The platform also includes visualisations for depicting time spent and energy expended within different intensity thresholds determined using metabolic equivalents (METs). These data are presented as minute-by-minute 24-hour line graphs (using a ‘heat’ colour palette) and are summarised into daily and weekly totals (Fig. 3). In order to convert energy expenditure to METs, age-specific equations are used to estimate Basal Energy Expenditure [35]. Activities with a MET value below 1.8 are considered as sedentary behaviour when using this specific monitoring technology [23, 36]. MET values greater or equal to 1.8 and less than 3 are considered to reflect light activity, while moderate and vigorous activities are calculated from MET values greater or equal to 3 and less than 6, and greater or equal to 6 [37]. Recently, evidence has emerged that short bouts of high-intensity exercise has profound metabolic and health benefits, e.g. short bouts of exercise at approximately 80–90 % maximal oxygen uptake [38]. In the absence of an accepted definition of such activity in free-living conditions, we define physical activity greater or equal to 10.2 METs as ‘highly vigorous’ [6]; equivalent to approximately 85 % maximal oxygen uptake in an average person [39].

**Fig. 4:**
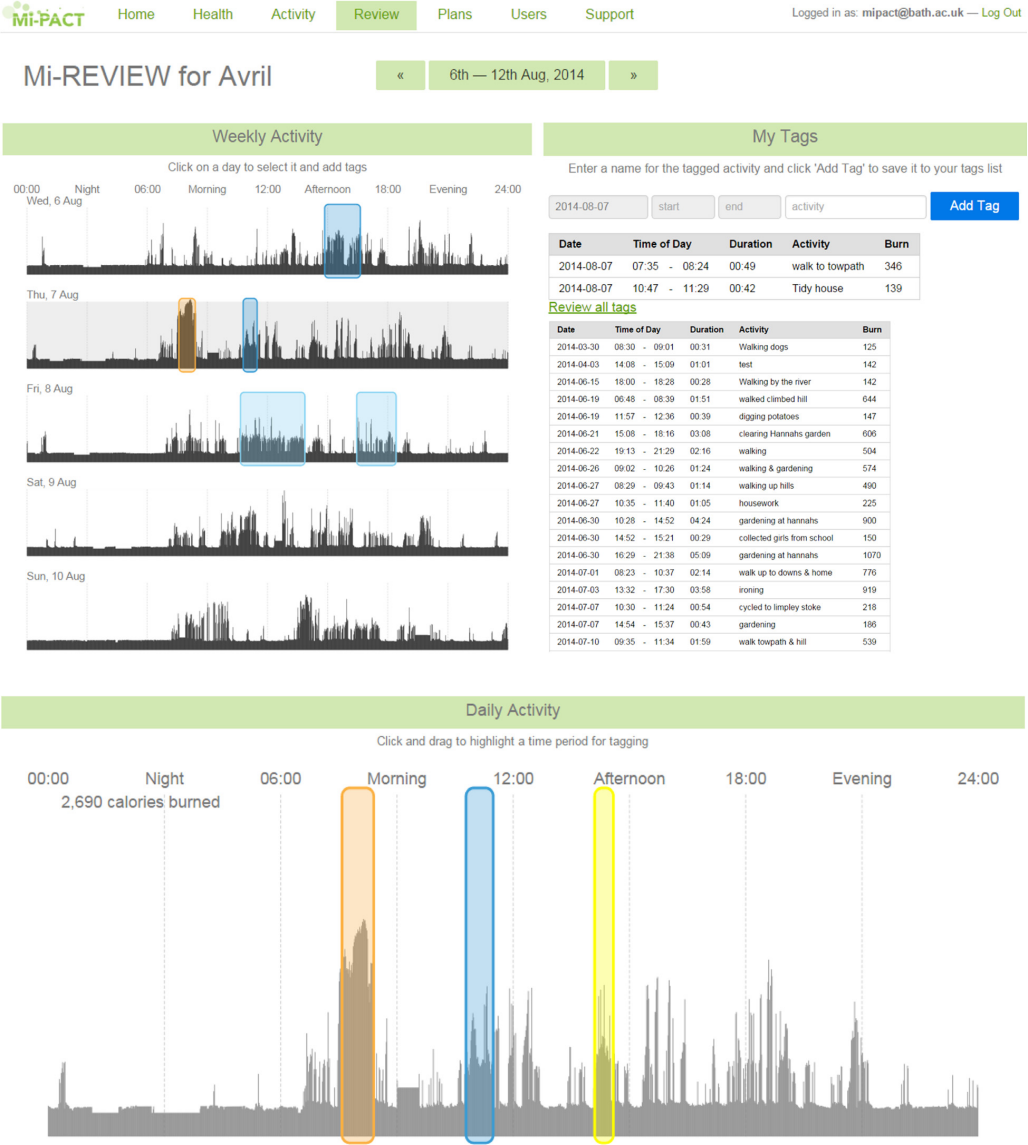
Platform screenshot displaying personalised minute-by-minute energy expenditure data and accompanying platform features for reviewing behaviour

**Fig. 5:**
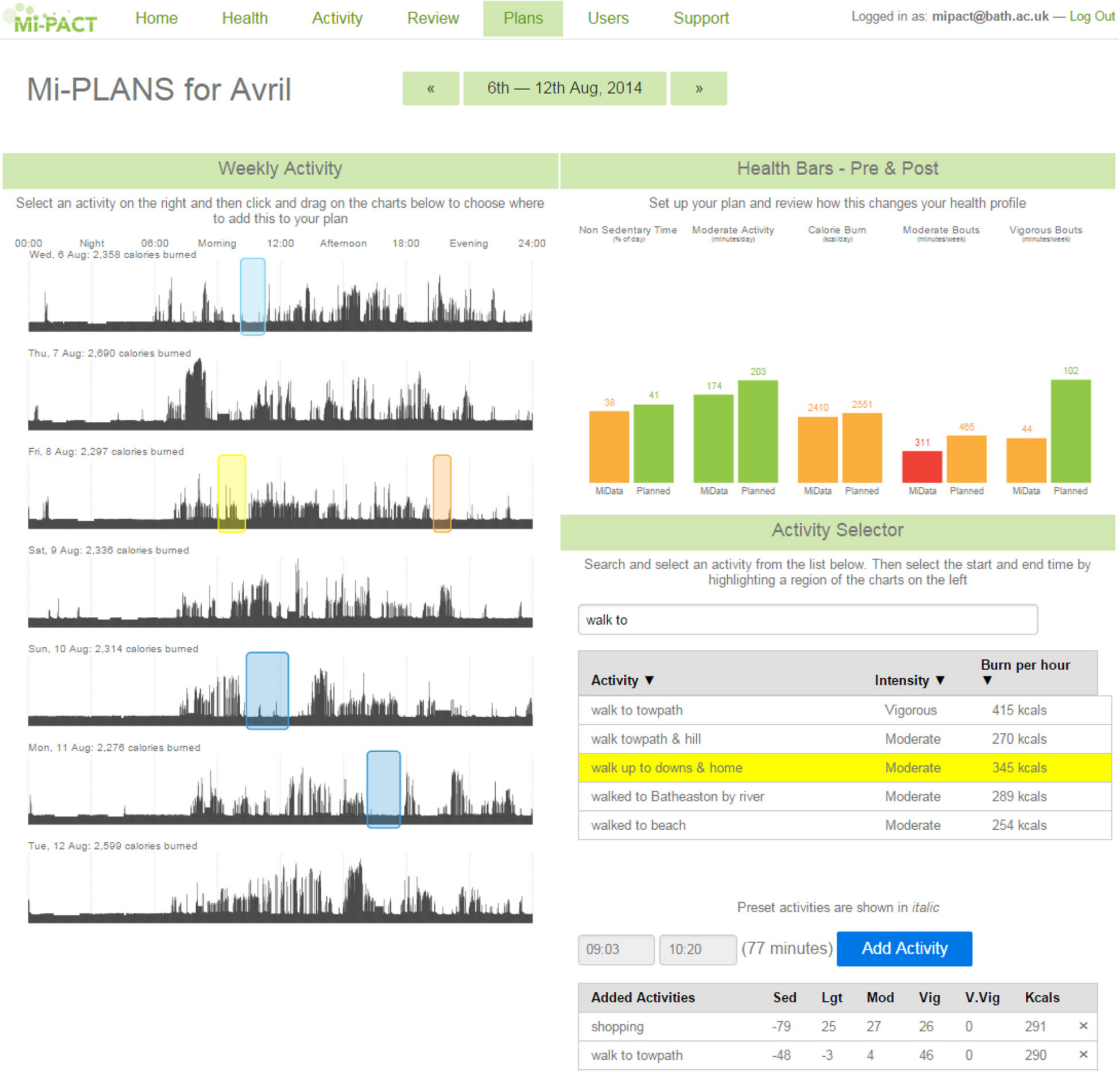
Platform screenshot depicting weekly activity patterns expressed relative to an individual’s multidimensional profile and features for exploring the impact of any changes and action plans on their behavioural goal(s)

In addition to providing feedback in the form of integrated physical activity profiles for ‘Health’ and as ‘Activity’ within different intensity thresholds, there are reviewing and planning components to the platform. The ‘Review’ section displays personalised minute-by-minute data (as a greyscale silhouette to emphasise activity patterning) that enables the individual to highlight, annotate or ‘tag’ and store information regarding discrete activities and behaviours as part of the self-monitoring process (Fig. 4). This ‘tagged’ information is then available as part of an individual’s historical data and viewable/editable on subsequent access of the platform. The ‘Plans’ section displays daily physical activity visualisations for the week and presents this information relative to the individual’s integrated physical activity profile (Fig. 5). In line with an implementation intention approach, there is an opportunity for the generation of specific plans regarding where, when and how to act. Indeed, the participant can explore the effects of exchanging sedentary behaviour for more positive behaviours (selecting from their personal ‘tags’ or from a database of activities) to realise the impact of any such substitution [8] on a change in their physical activity profile.

#### Intervention: Multidimensional individualised Physical ACTivity (Mi-PACT)

In the Mi-PACT group, feedback in the form of personalised physical activity profiles will be marketed, explained and discussed with participants in supportive one-to-one coaching sessions using the web-based platform. Participants will attend their GP practice for 5 consultations with a lifestyle coach: at baseline, and after approximately 2, 4, 8 and 12 weeks. Each participant will have access to activity monitors and the web-based platform for the duration of the 3-month intervention. Training materials for lifestyle coaches as well as hand-outs provided to study participants are available on request [40].

In the first session, participants will initially receive the same standardised messages as in the control group. The primary aim of this session is to explain the multidimensional nature of physical activity, to provide an understanding of which personalised behaviours have contributed to each dimension and to explain the options and choices that are available. A further objective is to familiarise the participant to activity self-monitoring and using the web-based platform. For this purpose, each participant will be given a Mi-PACT platform user manual and quick-start guide along with an activity monitor and Universal Serial Bus (USB) cable (for docking to their computer). Participants will have access to a personalised website account (protected by a log-in and password) and accompanying software for uploading their physical activity data. Prior to their next session, participants will be encouraged to experiment and engage in new and enjoyable activities (i.e. ‘trying different things’) while self-monitoring and exploring the functionality of the platform.

Subsequent sessions will be approximately 20–30 minutes in duration. Prior to meetings, coaches will be encouraged to log-in to the platform and review participants’ profiles as a preparatory exercise to help inform session delivery. Once it is established that the participant is confidently self-monitoring and reviewing their data, the aim of the second session is to revisit the participants’ physical activity profile and to discuss aspects of their physical activity behaviour that they would consider changing. Here, there will be a particular emphasis on identifying opportunities for achievable but sufficiently meaningful modification and specific goal setting regarding what, where and how to act. Participants who have low activity in every dimension have the most ‘choice’ and will be guided through a menu of physical activity options. Participants who score well in one or more dimensions will be supported in adding to their existing behaviour knowing that what they are doing is recognised. In terms of the tone of advice provided, coaches will attempt to use neutral language and encourage choice (e.g. using terms such as ‘you may choose to’ or ‘how would you’ rather than ‘you should’ or ‘you must’) and show empathy (e.g. ‘I appreciate why you might find this difficult’). These vocalisations will be provided in a structured manner such that goals, strategies, and implementation intentions are clear, realistic, and well-defined [10].

The primary focus of subsequent sessions will involve reviewing the impact of any changes and in supporting all efforts the participant has made to be more active, as well as recalibrating specific goals and plans within the context of the individual’s existing behaviour and lifestyle. This process will be led by the participant and will inevitably be highly individualised. At the final session, participants will be encouraged to consider their progress by using the multidimensional profile to reflect on what has been achieved and to develop a future action plan towards long-term change.

Participants will be urged to make the most of the web-based platform by wearing their physical activity monitor as much as possible (day and night) and to regularly upload their data and review their informational feedback over the 3-month intervention period. As the server has two portals, one for the participant and one for the research team, technical issues (e.g. with uploading data) can be identified and resolved by the research team. For participants without access to a computer or the Internet, coaches will facilitate the upload of data to the platform within session and the individual’s profile will be made available to them as a colour print-out where applicable. Participants with limited or no access to the platform outside of sessions will be provided with a diary for recording what aspects of their physical activity they have consciously changed between sessions (i.e. for self-monitoring purposes).

#### Training of the lifestyle coaches: intervention and control

We will recruit 4–6 intervention providers from the local community (including healthcare providers and other appropriate settings) with experience and qualifications as physical activity or lifestyle advisors. Intervention providers will be added to the GP’s office for the intervention period and may include registered healthcare professionals (e.g. dieticians) and/or non-registered professionals (e.g. health trainers). This mix of personnel is included in order to make the study as pragmatic and generalisable as possible to routine healthcare practice. To ensure delivery is compliant to treatment protocol, we will implement a number of strategies to maximise and monitor trial fidelity. Lifestyle coaches will participant in 2 days of training, including: (i) understanding the multidimensional nature of physical activity, (ii) familiarising with the web-based platform and self-monitoring technology, (iii) using the integrated physical activity profile for setting specific goals and forming implementation intentions, and (iv) provision of information in an autonomy-supportive manner (facilitating the participant’s satisfaction of autonomy and competence inherent within multidimensional profiling). All coaches will be given a written manual to support delivery of the intervention and will have the opportunity to practice and receive feedback on delivery style and content. The manual includes reference to general information on facilitating behaviour change adapted from the Department for Health NHS Health Trainer Handbook [41]. Whilst sessions are not specifically founded on Self-Determination Theory, they are designed to be delivered in an autonomy-supportive style. Other materials to further support delivery of and document the intervention will be produced as required.

#### Measurements

The primary outcome measure is physical activity, which will be directly assessed using physical activity monitors (see Physical Activity Assessment) for a 7-day period at all assessment points. We will use the underlying raw data for minute-by-minute physical activity energy expenditure to extract multiple physical activity characteristics and determine the change in a given (multiple) dimension(s) of physical activity behaviour. Specifically, this includes: overall energy expenditure (expressed as PAL); time engaged in moderate-to-vigorous physical activity accumulated on a minute-by-minute basis and in bouts of at least 10 minutes; time engaged in vigorous intensity activity accumulated in bouts of at least 10 minutes; and, non-sedentary time. To explore if the change in physical activity is meaningful, we will include secondary measures of weight, height, waist circumference, fat mass, lipids (total cholesterol, high-density lipoprotein and low-density lipoprotein cholesterol, and triglycerides), glucose control (insulin and glucose) and C-reactive protein. In addition, processes of change (e.g. need satisfaction and markers of internalisation), habit strength, motivation (autonomous and controlled), and indices of well-being, health status and quality of life will all be assessed. Together with physical activity data, these measures will allow us to examine whether changes in specific dimensions of physical activity are more or less important for specific outcomes (e.g. PAL, time > 3 METs). Based on previous research, small groups of 12–20 people in each physical activity category would be sufficient to determine whether a change in a particular aspect of physical activity was important for these secondary outcomes [42].

#### Anthropometry, blood pressure and body composition

Participants will be requested to remove any footwear and to wear only light clothing for anthropometric measurements. Body mass will be measured on a calibrated electronic or balance scale to the nearest 0.1 kg, where participants will be asked to stand in the centre of the platform with their weight evenly distributed on both feet. Height will be measured using a stadiometer to the nearest 0.1 cm. Participants will be requested to hang their arms freely with their heels, gluteal area and shoulders in contact with the stadiometer and with their head in the Frankfort plane (orbitale and tragion are horizontally aligned). The participant inspires for measurement, and the recorder brings down the headboard to compress the hair. From these collective measurements, BMI will be determined (kg.m^−2^). To assess waist circumference, participants will be asked to remove or lift their top to allow access to the measurement site. Otherwise, the measurement will be taken over the thinnest layer of clothing. With the participant standing with their feet together and weight evenly distributed, waist circumference will be assessed by positioning an anthropometric tape midway between the uppermost border of the iliac crest and the lower border of the costal margin, with the tape placed around the abdomen at the level of the midway point. Following a deep inhalation and a gentle expiration, the measurement is taken at the end of the expiration, with the tape snug but not compressing the skin. Three consecutive measurements will be made to the nearest 0.1 mm. Blood pressure will be assessed using a stethoscope or automated monitor after at least a 5-minute period of seated rest. With the arm supported at the level of the heart the measurement will be taken from the brachial artery on three consecutive occasions (with the lower of the last two measurements being recorded).

#### Dual energy X-ray absorptiometry

Total percentage body fat will be estimated using dual energy X-ray absorptiometry (in participants who choose to opt-in for this measure). Descriptive information for each participant including date of birth, height and weight will be entered into the software (Hologic, Bedford, UK) before they are asked to lie supine on the scanning table (Discovery, Hologic, Bedford, UK). Participants will be positioned centrally with feet equally spaced and arms with an even gap from the trunk and asked to remain as still as possible during the 7-minute scan. Following completion of the scan, whole body composition analysis will be performed with regions sectioned as recommended (Hologic, Bedford, UK). ‘Central adipose tissue’ (abdominal subcutaneous and visceral adipose tissue) will be estimated from a central region between L1–L4, which has previously been shown to correlate with measures of metabolic health [43]. Following an overnight fast, participants will be required to consume 1 pint of water on waking (to ensure adequate hydration) and to void their bladder prior to assessment. This is important to minimise variations in hydration status between individuals and because body water affects lean mass estimates [44]. In addition, participants will only wear light clothing for the analysis. Fat mass index (FMI) will be calculated using the equation:

$$ FMI = \mathrm{total}\ \mathrm{fat}\ \mathrm{mass}\ \left(\mathrm{kg}\right)/\mathrm{heigh}{t}^2\left({m}^2\right) $$

with participants classified according to FMI reference ranges for obesity classification [45].

#### Blood sampling and analysis

Fasting blood tests will take place at the GP practice between 08:00 and 11:00. Participants who are not fasted on arrival will complete the venepuncture on another occasion. Blood samples will be drawn by the research or practice nurse from an antecubital vein and dispensed into vacutainer collection tubes (Becton Dickinson, Oxford, UK) containing the anticoagulants ethylenediaminetetraacetic acid or fluoride oxalate for plasma samples, and serum-separator tubes for serum samples. Blood samples will be marked with a project identifier code and participant study number before being sent to the Royal United Hospital Bath NHS Trust Clinical Pathology Laboratory. Samples will be centrifuged at ambient temperature within 6 hours of collection (3120 g for 10 minutes). Samples will be separated and analysed within 24 hours (except for insulin which was frozen at −20 °C and analysed at a later date). Serum triglycerides, total and high-density lipoprotein cholesterol, plasma glucose, and C-reactive protein will be determined using a Cobas 8000 (Roche Diagnostics Limited, Burgess Hill, UK). Low-density lipoprotein cholesterol will be calculated using the Friedewald equation [46]. Insulin analyses will be undertaken on a Roche E170 analyser (Roche Diagnostics, Mannheim, Germany). The assay employs a direct electro-chemiluminescence immunoassay utilising a mouse monoclonal antibody labelled with ruthenium and a second mouse monoclonal antibody coupled to paramagnetic particles.

#### Questionnaire and qualitative measurements

The Psychological Need Satisfaction in Exercise Scale will be used to measure perceived competence, relatedness and autonomy [47] while motivational regulations for exercise will be assessed using the BREQ-2 [48]. Leisure-time physical activity habit will be measured using a four-item automaticity subscale of the Self-Report Habit Index [49], vitality using the Subjective Vitality Scale [50] and competence using the Perceived Competence Scale [51]. In addition, subscales from the Intrinsic Motivation Inventory [52, 53] will be adapted to specifically measure the dimensions of exercise-related interest/enjoyment, effort/importance and pressure/tension. Health status and quality of life will be assessed using the SF-36 Health Survey Questionnaire [54] and the EQ-5D 3 L [55]. In addition, the Department of Health’s Life-Stage Segmentation Toolkit [56] will be used to segment participants factoring in attitudinal and psychographic data (a person’s overall approach to life, including personality traits, values and beliefs) and within the context of their social and material circumstances.

We will conduct focus groups at the end of the trial with 20–30 participants from a subset of the intervention arm to explore their experiences from participating in the trial and to seek detailed feedback for the suitability and acceptability of the intervention components. These focus groups will specifically focus on the importance of the physical activity profiles, the usefulness of the meetings, the barriers and facilitators of change, the dimensions of physical activity that participants modified and any other lifestyle behaviours influenced by participation in the trial. We will employ purposeful sampling procedures to capture diverse experiences of responders and non-responders across different physical activity dimensions.

#### Physical activity assessment

Physical activity energy expenditure will be estimated using a Bodymedia Core monitor (BodyMedia Inc., Pittsburgh, PA, USA). This is a wireless multisensor device worn over the triceps muscle that integrates accelerometry and heat-related measurements (heat flux, galvanic skin response, skin temperature and near body ambient temperature), and sex, age, height and body mass to estimate energy expenditure using proprietary algorithms (SenseWear® Pro 8.0, algorithm v5.2, BodyMedia Inc., Pittsburgh, PA, USA). The monitor has rechargeable batteries and can be used to collect and store data for 2 weeks – with data sampled at 1-minute intervals. Previous research has shown the Bodymedia SenseWear® (BodyMedia Inc., Pittsburgh, PA, USA) device accurately measures energy expenditure relative to criterion measures [57–59] and has been increasingly used to quantify sedentary time, physical activity and energy expenditure in experimental trials [60, 61]. Participants will be required to wear the monitor for at least 6 days (including a Saturday and Sunday) to be included in the analysis [23], and will be instructed to only remove the device for showering and water-based activities. A valid day requires at least 80 % data for a given 24-hour period. As described previously, minutes spent in the distinct intensity thresholds based on metabolic equivalent cut points and multidimensional health target attainment will be calculated [6]. Data gaps will be assigned estimated basal energy expenditure [35].

#### Sham activity monitoring

A potential threat to any study that measures physical activity behaviour is confounding due to a Hawthorne effect (i.e. changes in behaviour that occur simply due to the special attention afforded by the intervention). In the past, we have deliberately avoided telling people that their physical activity is being observed (e.g. [7]) but, inevitably, this is not an option in the present study because the intervention group will become necessarily more aware of their physical activity and could potentially change their behaviour during specific outcome assessment periods (e.g. for social desirability). One approach to overcome this threat and to avoid any short-term changes in behaviour associated with physical activity outcome assessment is to extend the perceived assessment window. In order to achieve this, we will employ the use of a sham physical activity monitor worn on the wrist in the 1-month prior to follow-up measurements (in both groups). In most cases we will employ a genuine sham (i.e. an empty shell) but at least 5 % of sham monitors will be real working units (MotionWatch 8, Cambridge Neurotechnology Ltd., Cambridge, UK). The motion watch can record and store data for up to 120 days with a 1-minute sampling frequency. Participants will be told that some devices will be recording and others may not.

#### Trial fidelity

We will include a range of the strategies outlined by the National Institute of Health Behaviour Change Consortium to maximize and monitor trial fidelity across the established five domains of Study Design, Training, Delivery, Receipt, and Enactment [62]. First, we will implement standardised training of intervention providers (see Training of the lifestyle coaches: intervention and control) and will train more coaches than needed to protect against dropout. Trial fidelity will be monitored by: selective recording of consultation meetings; fidelity checklists (administered at the end of each session); and, provision of formative feedback to health trainers. Engagement of participants with the on-line platform will be assessed quantitatively by the number of occasions that participants logged in to the website. In addition, adherence to coaching sessions will be assessed by coach records of the number and format of support sessions delivered.

#### Concomitant medication and usual care

All changes in medications or new diagnoses during the course of the study will be recorded by the research nurse either in-clinic or following examination of patient records at baseline, 3 and 12 months. All relevant over-the-counter or prescription medication, the generic name of each medication, dose, frequency and history will be recorded. In addition, the research nurse will document any care participants receive in relation to supporting changes in weight or physical activity throughout the study.

### Statistics

#### Number of participants

Although an exploratory trial, it is prudent and instructive to conduct a formal sample size estimation. For this purpose, our specific outcome is mean physical activity energy expenditure (expressed as PAL). In middle-aged men [4], the standard deviation for PAL was 0.18 (we have no reason to suspect a substantially different variability in women). Our targeted effect size is a difference between intervention and control arms in the 12-month change in PAL from baseline to follow-up of 0.07. Based on our previous data [4], an increase in PAL of that magnitude would result in an increase of 10 % in the proportion meeting the minimum physical activity recommendation of a PAL of 1.6 [63], which we define as the smallest worthwhile effect [64]. With 2*P* = 0.05, 90 % power, and an assumed correlation between baseline and follow-up values of *r* = 0.7, the required sample size with an Analysis of Covariance model [65] is 108 in the intervention and 54 in the control. Allowing for 25 % loss to follow-up (attrition) results in a final target sample size of 144 in the intervention group and 72 controls. The assumed correlation between baseline and follow-up (the 12-month reliability) is a conservative estimate, as we are aware of no long-term reliability data using the best objective measures. Note that we are not, a priori, attaching primacy to PAL and we will examine multiple physical activity dimensions. PAL permits a robust sample size estimate and is clearly important.

#### Data analysis

Analyses will be undertaken on an intention-to-treat basis. The statistical analysis plan is for a primary comparison between intervention and control arms of the 12-month change in physical activity using an analysis of covariance model [66], with baseline values as the covariate to control for chance imbalances at baseline (accounting for any unequal variance due to the unequal allocation, and including the factors used in minimisation [67]). Analysis of interaction effects involving age-group or sex will be exploratory only and used to inform sample size planning for any subsequent definitive trial or evaluation. The analysis adopts a linear mixed model to provide the mean intervention effect together with quantification (as a standard deviation) of the individual participant differences in response to the intervention (‘treatment heterogeneity’) [68]. Confidence intervals, confidence levels, and magnitude-based inferences will be used to assess the clinical significance of the effect [64, 69, 70]. The same analysis strategy is applied to each dimension of physical activity, with due account taken of multiplicity [71]. In accordance with our second objective, for body weight and composition and metabolic control outcomes we will explore – using accepted regression-modelling methods – the extent to which intervention effects are mediated by changes in physical activity dimensions. Qualitative data will be analysed using thematic analysis based on constant comparison methods [72]. Techniques to enhance dependability and trustworthiness will include the development of a detailed coding scheme and coding checking protocol, cross-tabulation, negative case analysis and respondent validation (both in situ and by inviting feedback summaries of analysed data) [73].

## Discussion

It is important that physical activity fulfils its potential in making an effective contribution towards the success of public health initiatives. This paper describes the protocol for the Mi-PACT randomised controlled trial which aims to modulate physical activity, generate meaningful weight loss and improve metabolic health in patients at risk of cardiovascular disease and diabetes mellitus recruited from primary care. It is increasingly apparent that there are a variety of dimensions to physical activity that are independently important and we need to harness this information to avoid public confusion and disappointment regarding efforts to change physical activity behaviour. In particular, the marketing of informational feedback and set of physical activity options is potentially a key step in supporting increased engagement and sustained lifestyle modifications.

With technological innovation, the capture and provision of multidimensional physical activity ‘profiles’ is increasingly accurate, precise and scalable. In the present trial, we will examine whether multidimensional physical activity feedback from wearable physical activity monitors and self-monitoring using an online platform can be successfully incorporated into existing healthcare provision; and whether this leads to a meaningful change in physical activity in patients at risk of chronic disease. We envisage direct potential application within the context of public health initiatives (e.g. NHS Health Check). In addition, multidimensional physical activity profiling could become an adjunct to other public health strategies in primary practice (e.g. weight loss or cardiac rehabilitation).

## Trial status

Enrolment into the study started on 13 May 2014. Recruitment is expected to be completed by 1 June 2015 and follow-up assessment in a further 12 months.

## Abbreviations

BMI: body mass index
CONSORT: Consolidated Standards of Reporting Trials
FMI: fat mass index
GP: general practitioner
ISRCTN: International Standard Randomised Controlled Number
MET: metabolic equivalent
Mi-PACT: Multidimensional individualised Physical ACTivity
NHS: National Health Service
PAL: Physical Activity Level
TIDieR: Template for Intervention Description and Replication
USB: Universal Serial Bus
